# Identification of the N-terminal transmembrane domain of StarD7 and its importance for mitochondrial outer membrane localization and phosphatidylcholine transfer

**DOI:** 10.1038/s41598-017-09205-1

**Published:** 2017-08-18

**Authors:** Yasuhiro Horibata, Hiromi Ando, Motoyasu Satou, Hiroaki Shimizu, Satomi Mitsuhashi, Yasuo Shimizu, Masahiko Itoh, Hiroyuki Sugimoto

**Affiliations:** Department of Biochemistry, Dokkyo Medical University School of Medicine, 880 Kitakobayashi, Mibu, Tochigi, 321-0293 Japan

## Abstract

StarD7 facilitates phosphatidylcholine (PC) transfer to mitochondria, and is essential for mitochondrial homeostasis. However, the molecular mechanism for PC transfer by protein remains poorly understood. Herein, we describe a putative novel transmembrane (TM) domain C-terminal to the mitochondria-targeting signal (MTS) sequence at the N-terminus of StarD7. The mature form of StarD7 is integrated and/or associated onto the outer leaflet of the outer mitochondrial membrane (OMM) in HEPA-1 and HepG2 cells. A truncated form of StarD7 lacking the TM domain is distributed in the inner space of the mitochondria, and cannot reverse mitochondrial abnormalities, such as complex formation and PC content, when re-expressed in *StarD7-*KO HEPA-1 cells. Re-expression of wild StarD7 can compensate these mitochondrial functions of *StarD7-*KO HEPA-1 cells. The precursor form of StarD7 is cleaved between Met^76^ and Ala^77^, and Ala^77^ and Ala^78^ in the TM domain to produce the mature form. These results suggest that StarD7 is anchored onto the OMM through its N-terminal TM domain, and the C-terminal START domain may extend into the cytoplasm and shuttle PC between the ER and OMM at the ER-mitochondria contact sites.

## Introduction

Organelle membranes are composed of a phospholipid bilayer and embedded proteins. These membranes have defined phospholipid compositions, and maintenance of the correct composition is critical for homeostasis of the organelles. Phosphatidylcholine (PC) is the most abundant phospholipid in mammalian cells. Several other phospholipids, such as phosphatidylethanolamine (PE), phosphatidylserine (PS), cardiolipin (CL), phosphatidylinositol (PI), and sphingomyelin are also important membrane constituents. The endoplasmic reticulum (ER) contains multiple biosynthetic enzymes that catalyze the *de novo* biosynthesis of phospholipids, sphingolipids, and sterols, and is the major site for the biosynthesis of most membrane lipids in mammalian cells^1, 2^. Therefore, the selective transport of newly synthesized lipids in the ER to other organelles is important for the maintenance and proper function of organelles.

There are several mechanisms for the intracellular transport of membrane lipids. One is the vesicular transport of budding vesicles from a donor compartment to an acceptor compartment. Although vesicular transport mediates the bulk transport of many types of lipid, there is increasing evidence that non-vesicular lipid transport mediated by lipid-transfer proteins (LTPs) is the major transport pathway for certain lipids. LTPs generally have specific lipid-binding domains capable of facilitating lipid exchange. Based on their sequence and structural similarity, LPTs have been divided into families such as PI-transfer protein (PITP), steroidogenic acute regulatory protein (StAR)-related lipid transfer (START) domain containing protein (StarD), glycolipid transfer protein (GLTP), and oxysterol-binding protein (OSBP)-related protein (ORP)^2^. These proteins extract a specified lipid monomer from the cytoplasmic face of the outer leaflet of the donor membrane and deliver it to the outer leaflet of the target membrane. In addition, recent studies have demonstrated that membrane contact sites formed by tethering two organelles greatly contribute to lipid exchange. Some lipids, such as cholesterol, can be exchanged spontaneously at these contact sites. However, specific LTPs accelerate lipid transfer between the membranes^3^. For example, ceramide transfer protein (CERT) and four-phosphate adaptor protein 2 (FAPP2) regulate ceramide and glucosylceramide transfer, respectively, at the ER–Golgi contact site^4, 5^, and ORP5 and ORP8 mediate PS and PI_4_-phosphate (PI_4_P) transfer at the ER-plasma membrane contact site^6^.

PC is the predominant phospholipid (40–50%) in mitochondria, followed by PE (30–40%), CL (5–15%), PI (2–9%) and PS (1%). Mitochondria contain sequential enzymes for the synthesis of PE, CL and PG, but not for PC and PS. Similar to the organelles described above, mitochondria form membrane contact sites with the ER. A number of studies have shown that these ER-mitochondria contact sites facilitate the transfer of both calcium and lipid between the organelles. PS, synthesized in the ER, is transported to mitochondria and used for the production of PE by PS decarboxylase in the inner mitochondrial membrane. In yeast, the ER-mitochondrial connection is mediated by a protein complex referred to as the ER-mitochondria encounter structure (ERMES)^7^. ERMES facilitates PS but not PE transfer from the ER to mitochondria^8^. In mammals, mitofusin 2 (MFN2)^9, 10^, glucose-regulated protein 75 (GRP75)^11^, mitochondrial fission 1 protein (Fis1)-B-cell receptor-associated protein 31 (Bap31)^12^, and protein tyrosine phosphatase interacting protein 51 (PTPIP51)-vesicle-associated membrane protein-associated proteins (VAPs)^13^ have been reported to tether the ER and mitochondria. In contrast to PE synthesis, mitochondria lack enzymes to synthesize PC and therefore PC must be imported from the ER or other PC-containing organelles.

In our previous study, we identified a novel pathway for the transport of PC into mitochondria mediated by the LPT StarD7^14^. StarD7 belongs to the START domain–containing family. Family members contain ~210 amino acid residues for binding to specific lipids, including phospholipids, sterols, and sphingolipids^15^. There are two variable forms of StarD7: StarD7-I, which contains a mitochondria-targeting sequence (MTS) at the N-terminus and a START domain at the C-terminus, and StarD7-II, originally reported as gestational trophoblastic tumor gene-1 (GTT1)^16^, which lacks the MTS. StarD7-I localizes in both mitochondria and the cytosol whereas StarD7-II localizes exclusively in the cytosol. We demonstrated that both StarD7-I and StarD7-II preferentially bind, extract, and transfer PC from the donor membrane to the acceptor membrane *in vitro*. We also found that the intracellular transport of exogenously incorporated fluorescent PC into mitochondria is increased when StarD7-I is overexpressed in HEPA-1 cells^14^. Recently, we demonstrated that a deficiency of StarD7 in HEPA-1 cells not only reduced the mitochondrial PC content, but also impaired the formation of cristae, impaired mitochondrial respiration, and decreased the cell proliferation rate. It was reported that knockdown of StarD7 altered trophoblast function and differentiation in JEG-3 cells^17^. In addition, the loss of StarD7 protein resulted in alteration of ER and mitochondria morphology in HepG2 cells^18^. Yang *et al*. recently reported that StarD7 is required for mitochondrial and epithelial cell homeostasis in the lung^19^. We therefore proposed that StarD7 is essential for maintaining the level of mitochondrial PC, as well as for maintaining mitochondrial function and morphogenesis^20^. However, little is known about the precise molecular mechanism of StarD7-mediated PC transfer to mitochondria.

In this study, we analyzed the intra-mitochondrial localization and topology of StarD7. We found that StarD7 contains a transmembrane (TM) domain C-terminal to the N-terminal MTS. An alkaline extraction assay, proteinase K protection assay, and immunocytochemistry demonstrated that StarD7 is integrated into the outer mitochondrial membrane (OMM) *via* its TM domain, and exposes its C-terminal START domain to the cytoplasmic face. These results suggest that StarD7 exchanges/shuttles PC between the outer leaflet of other organelles such as the ER and the outer leaflet of the OMM at membrane contact sites.

## Results

### StarD7 is integrated into the mitochondrial membrane

**Figure 1:**
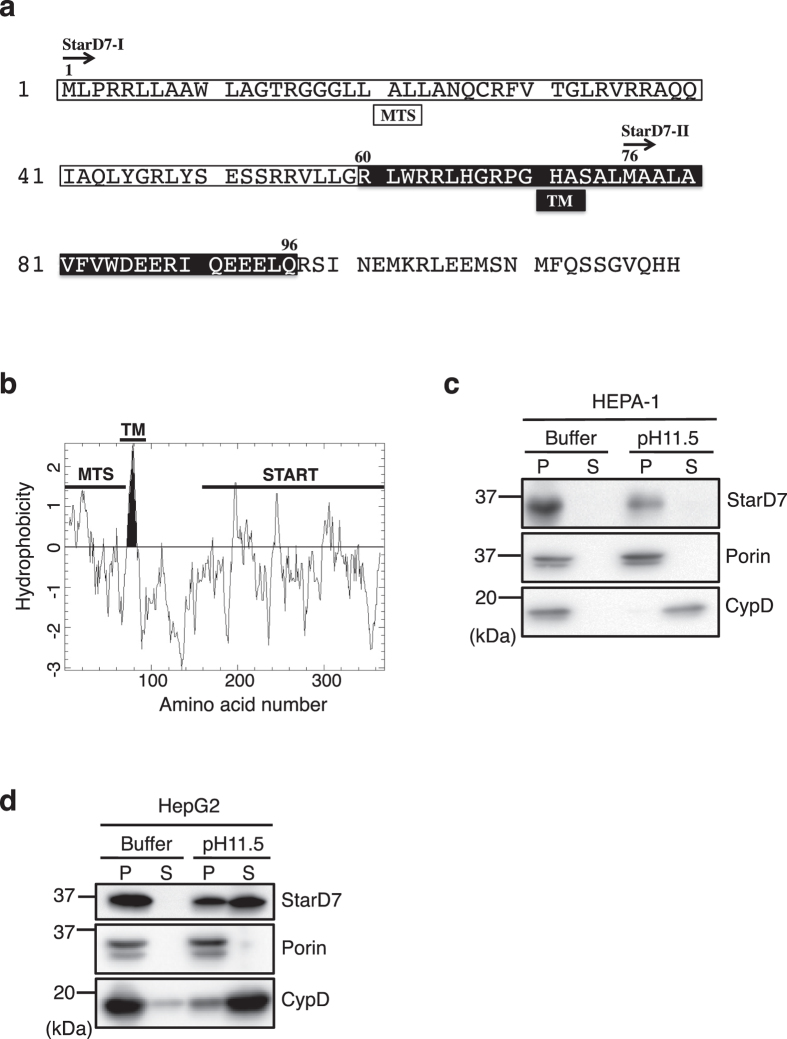
Endogenous StarD7 in HEPA-1 cells and HepG2 cells is integrated into the mitochondrial membrane. (**a**) The N-terminal amino acid sequence of StarD7. StarD7-I and StarD7-II start from Met^1^ and Met^76^, respectively. The putative mitochondrial-targeting signal (MTS) (Met^1^-Gly^59^) is *boxed*. The putative transmembrane (TM) domain (Arg^60^-Gln^96^) is indicated by *white letters in the black box*. (**b**) Hydrophobicity plot of human StarD7 as determined by Kyte-Doolittle prediction. The putative TM domain is indicated by *shading the peak black*. (**c** and **d**) Alkaline extraction assays for HEPA-1 (**c**) and HepG2 (**d**) cells. Mitochondria isolated from the cells were treated with isotonic buffer (Buffer) or alkaline buffer (pH 11.5), and then the pellet (P) and supernatant (S) were separated by centrifugation. The membrane and soluble fractions were dissolved in the same volume of sample buffer, and the same volume of protein solutions were separated by SDS-PAGE and analyzed by Western blotting with anti-StarD7, porin, and CypD antibodies. Porin is a membrane-integrated protein and CypD is a matrix protein.

Figure 1a shows the N-terminal amino acid sequence of human StarD7. StarD7-I is translated from the first Met, and has a MTS (Met^1^-Gly^59^) at the N-terminus. We previously demonstrated that StarD7-I is distributed in both mitochondria and the cytoplasm. In contrast, StarD7-II, originally reported as GTT1 by Durand *et al*.^16^, is translated from Met^76^ and is distributed exclusively in the cytosol. The examination of protein localization in our previous studies and in the present investigation confirmed that endogenous StarD7 is distributed in both mitochondria and the cytoplasm in HEPA-1 cells, rat liver^14^, and in HeLa and HepG2 cells (Fig. S1). These results suggested that StarD7-I is mainly expressed as endogenous StarD7 in these cells and rat liver tissue, and also probably in other cells and tissues. In this paper, unless otherwise specified, we refer to StarD7-I as StarD7.

We analyzed the primary structure of human StarD7 using a Kyte-Doolittle hydrophobicity plot^21^ and identified a highly hydrophobic region between amino acids Arg^60^ and Gln^96^, which is likely a TM domain (Fig. 1a and b). We also examined the hydrophobicity of amino acid sequences of StarD7 isolated from a variety of animal species. The putative TM domain appears to be conserved not only in mammals, but also in frog, fish, and invertebrates such as fruit fly and nematode (Fig. S2).

To confirm whether endogenous StarD7 is a membrane-integrated protein, we purified mitochondria from HEPA-1 cells and treated them with a high pH solution (pH 11.5) to release matrix proteins and membrane-associated proteins from the mitochondrial membrane. Following alkaline carbonate extraction, the pellet (membrane–integrated fraction) and supernatant (matrix and membrane-associated fraction) were separated by centrifugation. As shown in Fig. 1c, cyclophilin D (CypD), a mitochondrial matrix protein, was recovered in the supernatant. In contrast, StarD7 was detected in the pellet, similarly to the membrane-integrated protein porin, suggesting that StarD7 is a membrane-integrated protein. We also conducted the same experiment on HepG2 cells. As shown in Fig. 1d, StarD7 was also recovered in the pellet. In contrast to the results for HEPA-1 cells, about twice the protein was recovered in the supernatant, suggesting that StarD7 was also distributed in the mitochondrial matrix or associated to membrane in HepG2 cells.

**Figure 2:**
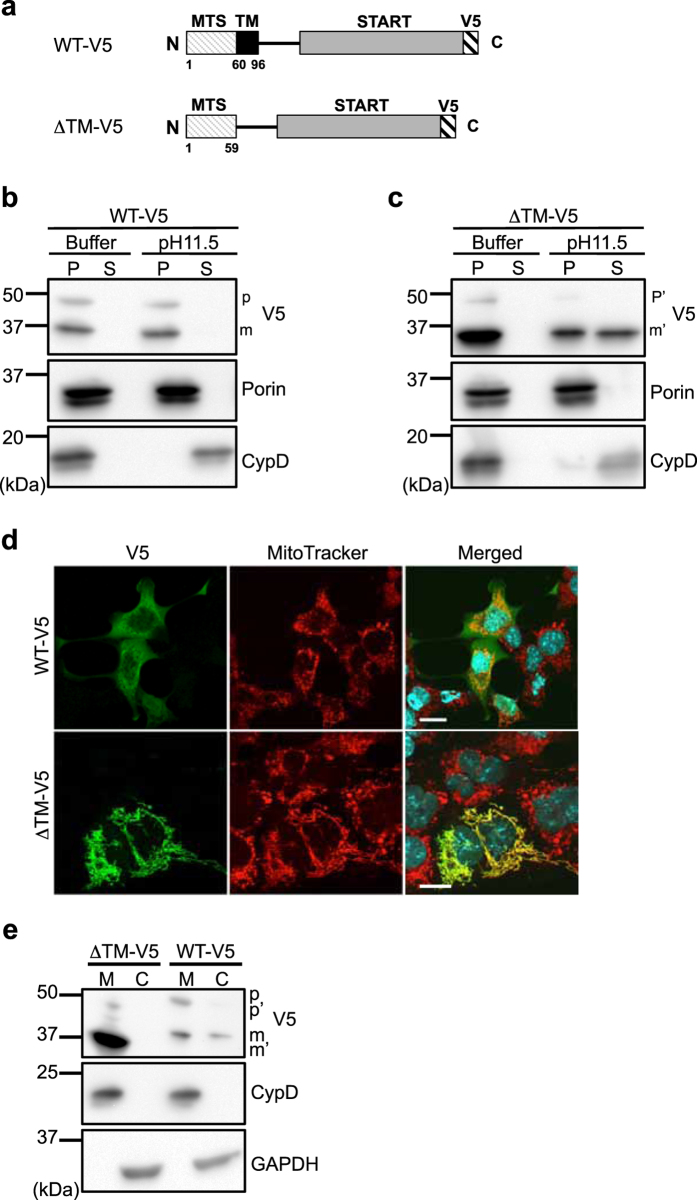
The TM domain of StarD7 is important for the integration of the protein into the mitochondrial membrane in HEPA-1 cells. (**a**) Schematic of WT-V5 and ΔTM-V5. The putative TM domain (Arg^60^-Gln^96^) was deleted in ΔTM-V5. V5-tags were fused at the C-terminus of both proteins. (b and c) Alkaline extraction assay. Mitochondria isolated from HEPA-1 cells transfected with WT-V5 (**b**) and ΔTM-V5 (**c**) were treated with isotonic buffer (Buffer) or alkaline buffer (pH 11.5). After centrifugation, the pellet (P) and supernatant (S) were dissolved in the same volume of sample buffer, and the same volume of protein solutions were analyzed by Western blotting using anti-V5, -porin and -CypD antibodies. p (48 kDa) and m (37 kDa), and p’ (45 kDa) and m’ (35 kDa) indicate the precursor and mature forms of WT and ΔTM protein, respectively. Porin is a membrane-integrated protein and CypD is a matrix protein. (**d**) HEPA-1 cells were transfected with the expression vector for WT-V5 or ΔTM-V5, then immunostained with anti-V5 antibody followed by anti-mouse IgG Alexa488 (green) and MitoTracker Red (red). Nuclei were stained with DAPI (blue). *Bars* indicate 10 μm. (**e**) Mitochondria and cytosol were separated from cells transfected with WT-V5 or ΔTM-V5 by subcellular fractionation. Proteins were analyzed by Western blotting using anti-V5, -CypD and -GAPDH antibodies. M and C indicate mitochondria and cytosol, respectively.

To assess whether the putative TM domain between Arg^60^ and Gln^96^ contributes to the membrane-integration of StarD7, we constructed a truncated protein (ΔTM-V5) that lacks the TM domain (Fig. 2a). V5-tags were fused at the C-terminus of both the wild type (WT) and mutated proteins. After transfection of these constructs into HEPA-1 cells, mitochondria were isolated from the cells, treated with the pH 11.5 solution, and the protein content was analyzed by Western blotting. As shown in Fig. 2b and c, two protein bands were observed in both the WT and mutated cell extracts upon analysis with anti V5-antibody. The upper band was a 48-kDa precursor (p) and the lower band was a 37-kDa mature form (m) processed by mitochondrial peptidase^14^. Both the p and m forms of WT-V5 were quantitatively recovered in the membrane-integrated fraction, similar to the results for endogenous StarD7 in HEPA-1 cells (Figs 2b and 1c). Following the expression of ΔTM-V5, both the precursor form (p’) and the mature form (m’) were detected (Fig. 2c). The molecular weight of the m’ form of ΔTM-V5 was slightly lower than that of wild-type StarD7, suggesting that their cleavage sites for mitochondrial peptidase are different. In contrast to WT-V5, a significant amount of ΔTM-V5 (m’) was detected in the supernatant of the ΔTM-V5 sample, suggesting that the TM domain plays an important role in the mitochondrial membrane-integration of StarD7. ΔTM was also recovered in the pellet, suggesting that the protein is associated with the mitochondrial membrane. We speculated that the ΔTM-V5 protein binds to membrane PC through a START domain, which can bind/transfer PC^14^.

These constructs were transfected into HEPA-1 cells and the localization of WT-V5 and ΔTM-V5 protein in the cells was evaluated by cell fractionation, and by immunocytochemistry using anti-V5 antibody. As we reported previously, WT-V5 was located in both the mitochondria and cytosol (Fig. 2d and e). In contrast, ΔTM-V5 was localized only in the mitochondria, suggesting that the TM domain is important for both membrane-integration and cytoplasmic localization.

### The TM domain is required for anchoring StarD7 onto the OMM

**Figure 3:**
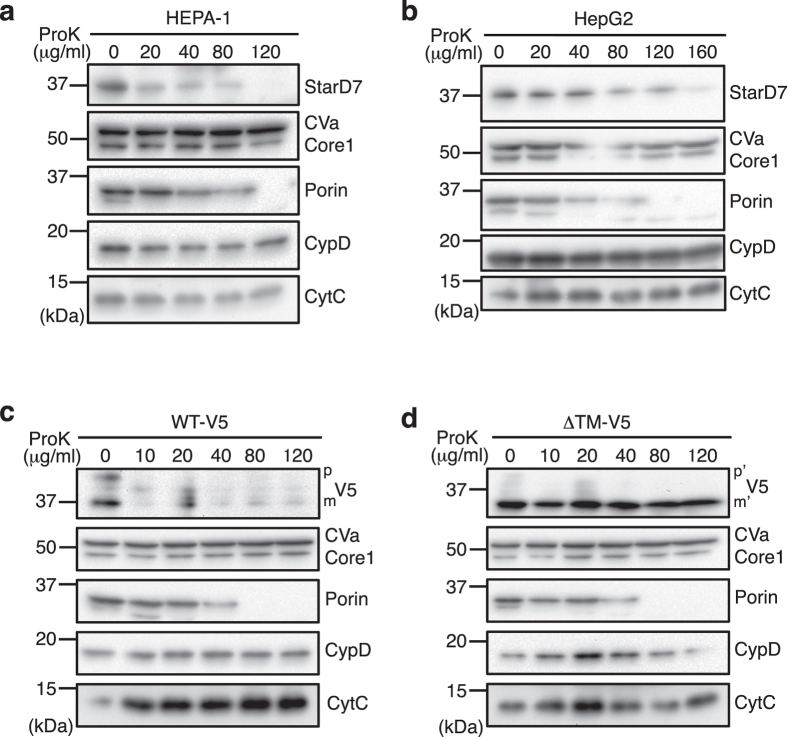
The TM domain is essential for the integration of StarD7 into the OMM. Mitochondria isolated from HEPA-1 (**a**), HepG2 (**b**), and HEPA-1 cells transfected with WT-V5 (**c**) or ΔTM-V5 (**d**) were treated with the indicated concentration of proteinase K (ProK). After separation by SDS-PAGE, endogenous or V5-tagged StarD7 were analyzed by Western blotting using anti-StarD7 antibody. (**a** and **b**) or anti-V5 antibody (**c** and **d**). Mitochondria marker proteins were analyzed using anti-CVa, -Core1, -porin, -CypD and -CytC antibodies. p and m (p’ and m’) indicate the precursor and mature forms of protein, respectively. Porin distributes in the outer mitochondrial membrane (OMM). CVa, Core1, CypD, and CytC are localized in the inner mitochondrial space.

In our previous study, we performed a proteinase K protection assay using mitochondria isolated from HEPA-1 cells, and demonstrated that endogenous StarD7 is anchored onto the OMM^14^. Here, we repeated this experiment using more precise localization marker proteins in the mitochondria. Purified mitochondria from HEPA-1 and HepG2 cells were treated with different concentrations of proteinase K, and StarD7 and other mitochondrial proteins were analyzed by Western blotting. As shown in Fig. 3a and b, endogenous StarD7 was entirely degraded in both HEPA-1 and HepG2 cells by treatment with proteinase K. Porin, a protein which distributes into the OMM, was also degraded, whereas there was no effect on intramitochondrial proteins such as complex Va (CVa), complex III Core1 (Core1), CypD, and cytochrome C (CytC). These results suggest that endogenous StarD7 in both cells is located on the OMM. It was also suggested that StarD7 recovered in the supernatant fraction in HepG2 (Fig. 1d) is associated onto the OMM.

We then performed the same experiment using mitochondria purified from HEPA-1 cells transfected with WT-V5 or ΔTM-V5. As shown in Fig. 3c, WT-V5 was sensitive to proteinase K, similar to endogenous StarD7. However, ΔTM-V5 was not degraded by proteinase K, demonstrating that ΔTM-V5 is located in the inner mitochondrial space. These results suggest that the TM domain plays an important role in anchoring StarD7 onto the OMM.

### The MTS-TM segment is important for the protein localization onto the OMM, but is not sufficient for release into cytosol

**Figure 4:**
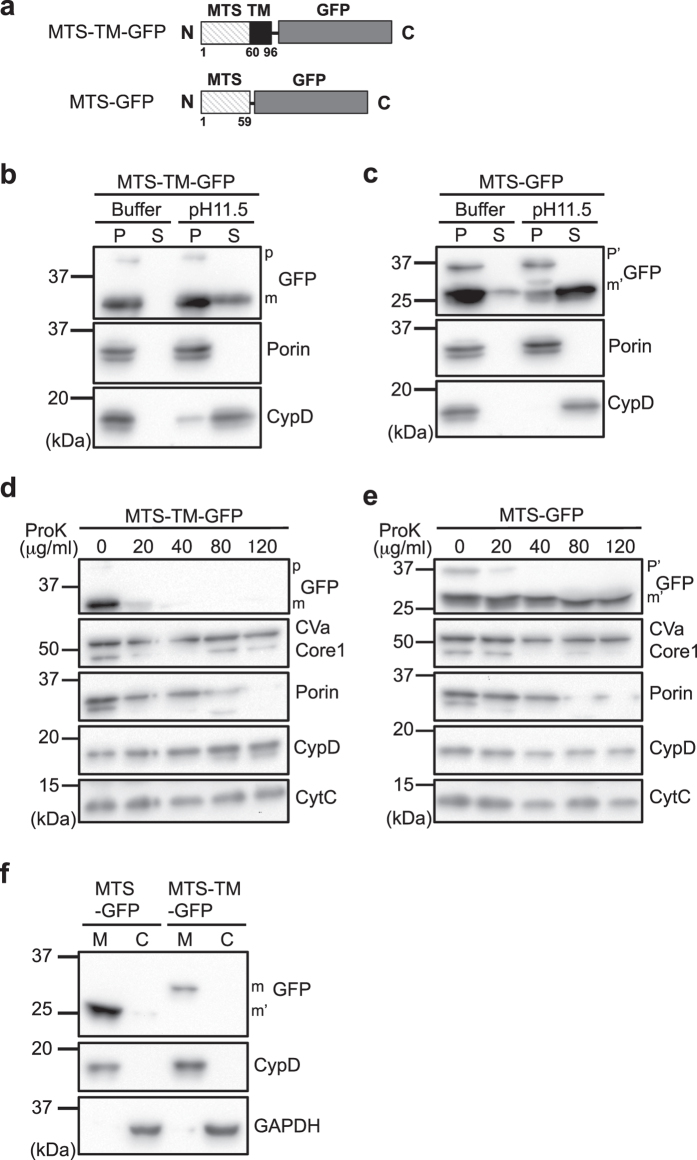
The MTS-TM segment in the N-terminus of StarD7 is sufficient for protein localization in the OMM. (**a**) Schematic of MTS-TM-GFP and MTS-GFP. In MTS-TM-GFP and MTS-GFP, MTS-TM and MTS are fused with GFP at the N-terminus, respectively. (**b** and **c**) Alkaline extraction assay. Mitochondria isolated from HEPA-1 cells transfected with MTS-TM-GFP (**b**) or MTS-GFP (**c**) were treated with isotonic buffer (Buffer) or alkaline buffer (pH 11.5), then the pellet (P) and supernatant (S) were separated by centrifugation, dissolved in the same volume of sample buffer, and the same volume of protein solutions were analyzed by Western blotting using anti-GFP, -porin and -CypD antibodies. (**d** and **e**) Proteinase K protection assays. Mitochondria isolated from HEPA-1 cells transfected with MTS-TM-GFP (**d**) or MTS-GFP (**e**) were treated with the indicated concentration of proteinase K (ProK). After separation by SDS-PAGE, GFP and mitochondrial marker proteins were analyzed by Western blotting using anti-GFP, -CVa, -Core1, -porin, -CypD and -CytC antibodies. p and m (p’ and m’) indicate the precursor and mature forms of the protein, respectively. (**f**) Mitochondria and cytosol were separated from cells transfected with MTS-GFP or MTS-TM-GFP by subcellular fractionation. M and C indicate mitochondria and cytosol, respectively.

We confirmed the importance of the MTS and TM domains at the N-terminus of StarD7 for anchoring the protein onto the OMM by constructing a chimeric green fluorescence protein (GFP) fused either with both MTS and TM (MTS-TM-GFP) or with MTS alone (MTS-GFP) at the N-terminus (Fig. 4a). After transfection of these constructs into HEPA-1 cells, the mitochondria were isolated, treated with the pH 11.5 solution, and protein distribution was analyzed by Western blotting using anti-GFP antibody. Similarly to the results obtained with V5-tagged StarD7, an upper band corresponding to precursor (p) and a lower band corresponding to the cleaved, mature form (m) were observed (Fig. 4b,c,d and e). More than half of the MTS-TM-GFP was detected in the pellet after the alkaline extraction assay, demonstrating that the protein is integrated into the mitochondrial membrane (Fig. 4b). Less than half of the protein was recovered into the supernatant, suggesting that it was also distributed in the mitochondrial matrix or associated onto membrane. The majority of MTS-GFP was recovered in the supernatant after the alkaline extraction assay, indicating that the protein was not integrated into the mitochondrial membrane (Fig. 4c). As compared to the results for ΔTM-V5 (Fig. 2c), considerably less MTS-GFP was recovered in the pellet after alkaline extraction assay. This supports our hypothesis that ΔTM-V5 is able to bind to the membrane through a START domain. Next, we treated the isolated mitochondria with protease K and analyzed protein integrity. MTS-TM-GFP was apparently integrated and/or associated to the OMM because it was entirely digested by protease K (Fig. 4d). However, as shown in Fig. 4e, MTS-GFP was not degraded by proteinase K, indicating that the protein was imported into the inner mitochondrial space. These results suggest that both the MTS and TM domains at the N-terminus are important and sufficient for anchoring the protein onto the OMM.

Next, we analyzed protein localization of chimeric GFP by subcellular fractionation. As shown in Fig. 4f, both MTS-TM-GFP and MTS-GFP were mainly recovered in mitochondria, but not in cytosol, suggesting that the MTS-TM segment is not sufficient for release from mitochondria to cytosol.

### Analysis of intra-mitochondrial localization of StarD7 by immunocytochemistry

**Figure 5:**
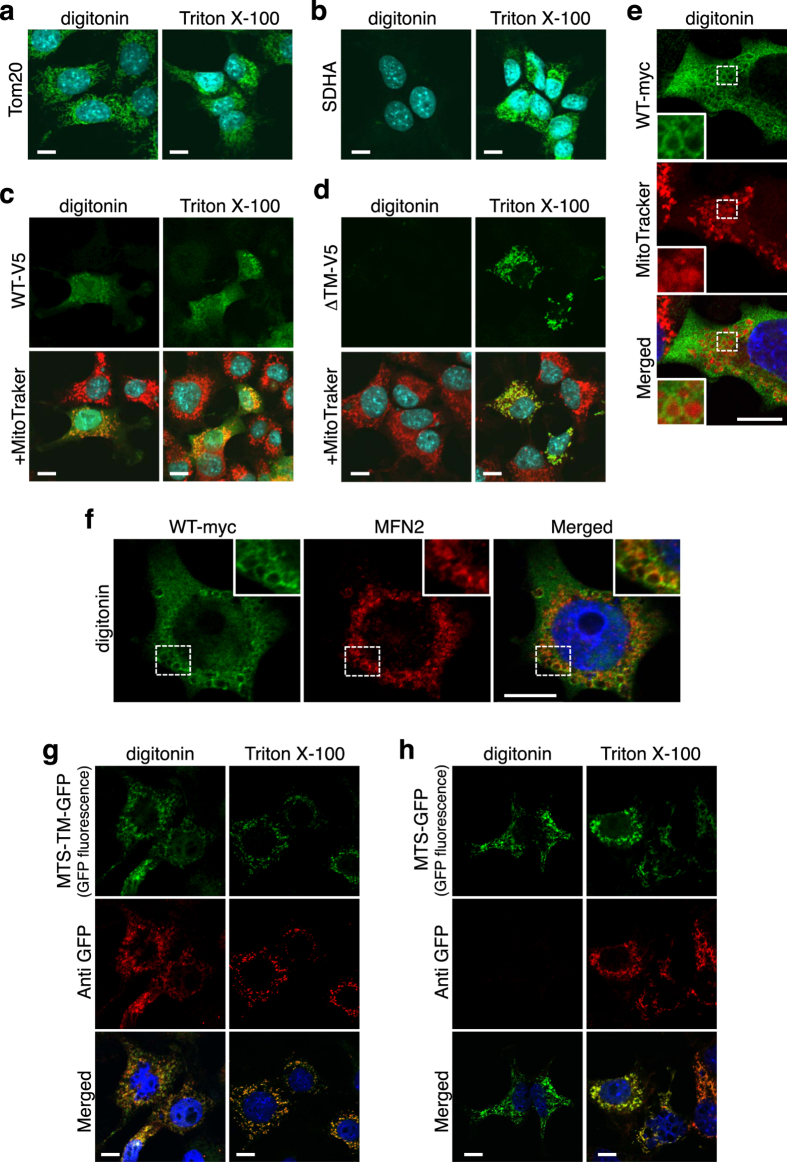
Immunocytochemical distribution of wild type or ΔTM StarD7 over-expressed in HEPA-1 cells. (**a** and **b**) HEPA-1 cells were permeabilized with 0.005% digitonin (w/v) or 0.1% Triton X-100 (w/v), then immunostained with anti-TOM20 (**a**) or –SDHA (**b**) antibodies followed by anti-mouse IgG Alexa488 (green). (**c**,**d** and **e**) HEPA-1 cells were transfected with the expression vector for WT-V5 (**c**), ΔTM-V5 (**d**), or WT-myc (**e**), permeabilized with 0.005% digitonin (w/v) or 0.1% Triton X-100 (w/v), then immunostained with anti-V5 or -myc antibodies followed by anti-mouse IgG Alexa488 (green) and MitoTracker Red (red). (**f**) HEPA-1 cells transfected with WT-myc were permeabilized with 0.005% digitonin (w/v) and immunostained using anti-myc and -MFN2 antibodies followed by anti-mouse IgG Alexa488 (green) and anti-rabbit IgG Alexa594 (red), respectively. (**g** and **h**) HEPA-1 cells were transfected with the expression vector for MTS-TM-GFP (**g**) or MTS-GFP (**h**), permeabilized with 0.005% digitonin (w/v) or 0.1% Triton X-100 (w/v), then immunostained with anti-GFP antibody followed by anti-mouse IgG Alexa594 (red). Nuclei were stained with DAPI (blue). *Bars* indicate 10 μm.

To confirm the results obtained from proteinase K treatment, indicating that StarD7 is anchored onto the OMM, we analyzed its localization by immunocytochemistry. HEPA-1 cells were treated with 0.005% digitonin (w/v) or 0.1% Triton X-100 (w/v) after fixation, stained with antibodies against translocator of the outer membrane20 (TOM20) or succinate dehydrogenase complex iron sulfur subunit A (SDHA), then the cells and their mitochondria were analyzed with a laser-scanning confocal microscope. As shown in Fig. 5a, 0.005% digitonin was able to permeabilize the plasma membrane but not the mitochondrial membrane, given that the mitochondria were stained with TOM20 whereas SDHA, which localizes in the mitochondrial matrix, was not stained (Fig. 5b). In contrast, 0.1% Triton X-100 was able to permeabilize both the plasma and mitochondrial membranes because both TOM20 and SDHA were stained (Fig. 5a,b). We then analyzed the intra-mitochondrial localization of WT-V5 and ΔTM-V5 using 0.005% digitonin (w/v) or 0.1% Triton X-100 (w/v). As shown in Fig. 5c, both mitochondrial and cytosolic WT-V5 was visualized in the digitonin-treated cells, suggesting that the protein is localized on the cytoplasmic face of the mitochondrial membrane. In contrast, ΔTM-V5 was stained in Triton X-100-treated cells, but not in digitonin-treated cells (Fig. 5d) due to its intra-mitochondrial localization.

We also expressed StarD7 fused to a myc-tag instead of the V5-tag at the C-terminus (WT-myc) in HEPA-1 cells to reduce both the background and non-specific signals compared to using V5-antibody. As shown in Fig. 5e, WT-myc clearly localized onto the OMM, as observed by staining the mitochondria with MitoTracker.

Mitochondria form membrane contact sites with the ER through membrane-tethering proteins such as MFN2. StarD7 distribution at ER-mitochondrial contact sites was investigated by permeabilizing HEPA-1 cells with digitonin, then analyzing the co-localization of StarD7 WT-myc and MFN2 in the cells. As shown in Fig. 5f, some myc-tagged StarD7 co-localized with endogenous MFN2, suggesting that StarD7 was distributed at ER-mitochondrial contact sites on the OMM.

We also analyzed the localization of chimeric GFP using anti-GFP antibody. As shown in Fig. 5g and h, anti-GFP antibody stained MTS-TM-GFP, but not MTS-GFP, in the digitonin-treated cells, indicating that chimeric MTS-TM-GFP exhibits the same behavior as V5-tagged StarD7. These results strongly support the results obtained from the proteinase K protection assay that StarD7 is anchored onto the OMM.

### The TM domain in StarD7 is essential for recovering the mitochondrial function and PC content in StarD7-KO cells

**Figure 6:**
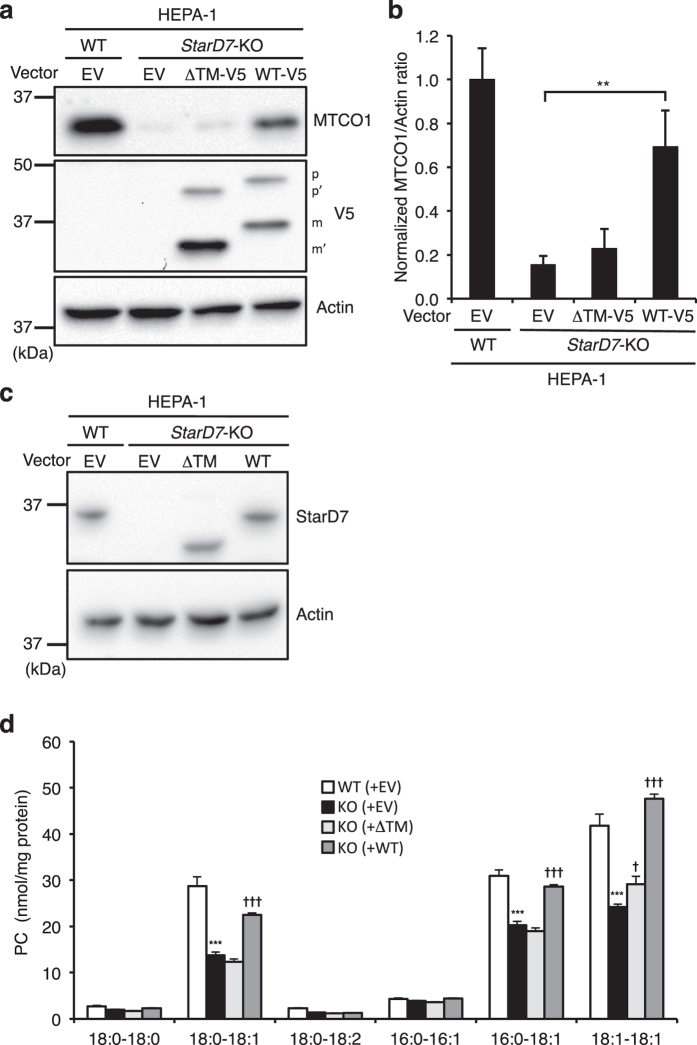
Membrane-integration *via* the TM domain is essential for maintaining the mitochondrial function of StarD7. (**a**) *StarD7*-KO HEPA-1 cells were transfected with empty vector (EV), WT-V5 or ΔTM-V5 and cultured for 3 days. After cell lysates (18 μg of protein) were separated by SDS-PAGE, MTCO1 and V5-tagged StarD7 were analyzed by Western blotting using anti-MTCO1 and -V5 antibodies. Actin was used as a protein loading control. (**b**) The density of the MTCO1 band was measured using Quantity One software and normalized against actin. Data were obtained at the linear range of signal intensity. Values are means ± S.D. from three independent experiments. **P < 0.01 as compared with KO cells with empty vector. (**c**) *StarD7*-KO HEPA-1 cells stably expressing EV, ΔTM or WT were established by G418 selection. Protein expression was analyzed by Western blotting using anti-StarD7 antibody. (**d**) After mitochondria were purified from the cells, phospholipids were extracted, and the amount of individual PC was determined using LC/MS/MS with multiple reaction monitoring. Values were normalized against protein amount of mitochondria. Data are means ± S.D. from quadruplet analyses in one experiment. ***P < 0.001 as compared with WT cells transfected with EV. ^†^ and ^†††^ indicate significant differences as compared to the KO cells transfected with EV (*P* < 0.05 and *P* < 0.001, respectively).

We previously established *StarD7*-KO HEPA-1 cells and demonstrated that the KO cells show several mitochondrial abnormalities, such as reduced mitochondrial PC and mitochondrially encoded cytochrome c oxidase 1 (MTCO1) content, and lower ATP synthesis and growth rate. We also found that some of these mitochondrial dysfunctions of *StarD7*-KO cells were restored when StarD7-I, but not StarD7-II, was transiently expressed. These results suggested that StarD7-II, a cytosolic form of StarD7, had lost the ability to maintain mitochondrial function, even though StarD7-II retained PC transfer activity *in vitro* ^14^. To reveal the role of the TM domain of StarD7 in maintaining mitochondrial function, we transfected WT-V5 and ΔTM-V5 into *StarD7*-KO cells. As shown in Fig. 6a and b, the reduced level of MTCO1 protein in the KO-cells was partially rescued upon over-expression of WT-V5, whereas the same effect was not observed when ΔTM-V5 was over-expressed. These results indicated that anchoring onto the OMM *via* the TM domain is essential for StarD7’s role in helping maintain regular mitochondrial function.

Next, we analyzed whether mitochondrial PC content could be restored when StarD7 is re-expressed in *StarD7*-KO cells. We established *StarD7*-KO cells stably expressing WT or ΔTM by G418 selection (Fig. 6c). Mitochondria were isolated using a Percoll/Nycodenz discontinuous density gradient, and the PC content was quantified by liquid chromatography-tandem mass spectrometry (LC/MS/MS). As shown in Fig. 6d, the major mitochondrial PC species, consisting of 18:0–18:1, 16:0–18:1, and 18:1–18:1 fatty acid moieties, were significantly reduced in *StarD7*-KO cells as reported previously^20^. However, the level of these PC species was significantly restored in *StarD7*-KO cells expressing WT, but not ΔTM. These results strongly suggest that membrane-integration through the TM domain is essential for the PC-transferring activity of StarD7.

### Identification of the StarD7 cleavage site for mature form production

**Figure 7:**
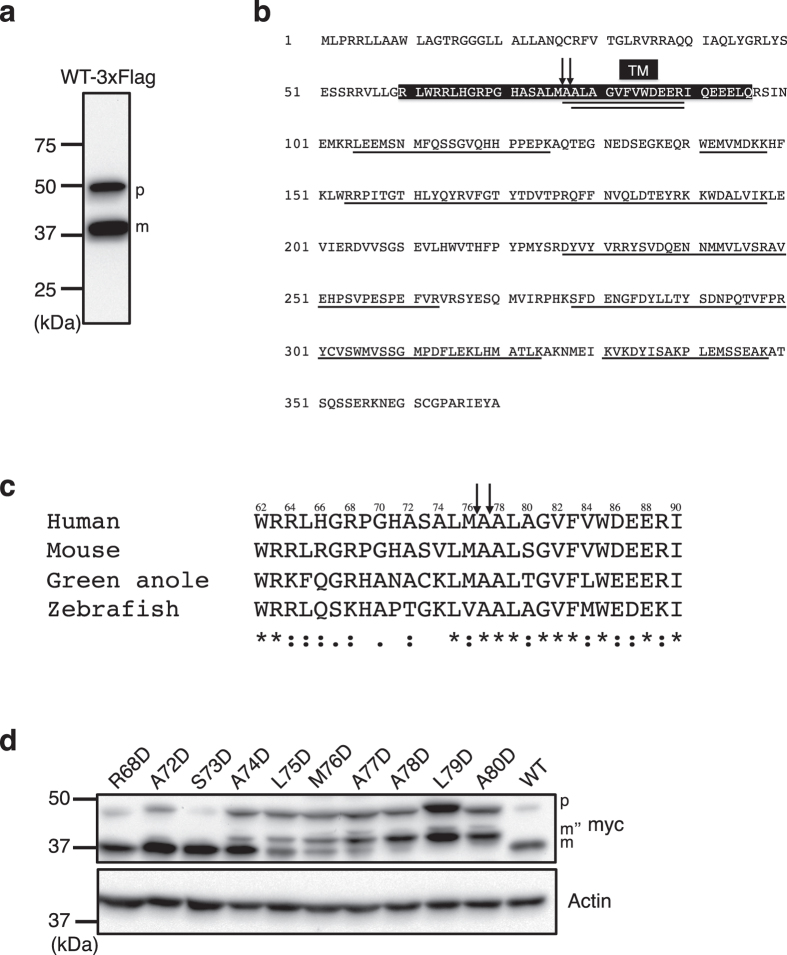
Determination of the cleavage site in the TM domain of StarD7 after integration into the mitochondrial membrane. (**a**) HEPA-1 cells were transfected with a plasmid encoding human StarD7 fused with the 3xFlag tag at the C-terminus. After lysing the cells, StarD7 was affinity purified using anti-Flag antibody conjugated to beads and analyzed with Western blotting using anti-Flag antibody. p and m indicate the precursor and mature forms of the protein, respectively. (**b**) The protein band corresponding to the mature form was excised and analyzed by LC/MS/MS. The identified sequences are *underlined*. The TM domain (Arg^60^-Gln^96^) is indicated by *white letters in a black box*. Arrows indicate possible cleavage sites. (**c**) Sequence alignment of StarD7 protein around the cleavage site in the TM domain. Asterisks indicate identical amino acids, and chemically similar amino acids are indicated by either *two dots* (very similar) or *one dot* (similar). (**d**) Amino acids around the cleavage site of StarD7 (Arg^68^, Ala^72^, Ser^73^, Ala^74^, Leu^75^, Met^76^, Ala^77^, Ala^78^, Leu^79^, or Ala^80^) were individually mutated to an aspartic acid, fused with myc-tag at the C-terminus, and expressed in HEPA-1 cells. Cell lysates were subjected to SDS-PAGE and immunoblotting using antibodies against myc and actin. m” and m indicate first-step and second-step cleaved StarD7, respectively. Actin was used as a protein loading control.

StarD7 is processed to produce the mature (m) form in mitochondria. To determine the cleavage site, StarD7 fused with the 3xFlag tag at the C-terminus was transiently expressed in HEPA-1 cells, then affinity-purified using anti-Flag antibody-conjugated agarose beads. The protein band corresponding to the mature form (m in Fig. 7a) was excised, digested with trypsin, and the peptide sequences were analyzed by LC/MS/MS. As shown in Fig. 7b, several tryptic peptides with a lysine or arginine residue at both the N- and C-terminal ends were identified. Of these, the AALAGVFVWDEER (Ala^77^-Arg^89^) and ALAGVFVWDEER (Ala^78^-Arg^89^) peptides (Fig. S3), those originate from the TM domain, are likely the N-terminus of the mature form because the N-terminal end of the peptides is neither a lysine nor an arginine residue. These results suggest that the peptide bonds between Met^76^ and Ala^77^, and Ala^77^ and Ala^78^ within the TM domain are cleaved by a mitochondrial peptidase to produce the mature form.

As shown in Fig. 7c, the amino acid sequence around the putative cleavage site in the TM domain is highly conserved, from mammals to fish. We conducted mutational analysis to confirm the contribution of these amino acids on proteolytic processing. Myc-tagged human StarD7 containing individual substitutions of conserved amino acids (Arg^68^, Ala^72^, Ser^73^, Ala^74^, Leu^75^, Met^76^, Ala^77^, Ala^78^, Leu^79^, or Ala^80^) to aspartic acid were constructed by site-directed mutagenesis, expressed in HEPA-1 cells, and protein processing was analyzed by Western blotting. As shown in Fig. 7d, R68D, A72D, and S73D mutants were processed to produce normal mature forms (m) similar to WT. Other mutants showed a significant accumulation of the precursor form (p), and were processed to yield mature forms (m”) exhibiting a higher molecular weight than that of the normal mature form (m). According to the results for A74D, L75D, M76D and A77D, it appeared that StarD7 is cleaved to yield m” by first-step cleavage, and then m” is further processed to yield m by a second-step cleavage event. For A78D, L79D and A80D, the second step of processing was fully blocked. These results suggest that StarD7 is cleaved twice by two separate proteolytic steps, and that the amino acid residues around the putative cleavage sites are critical for proper cleavage.

## Discussion

**Figure 8:**
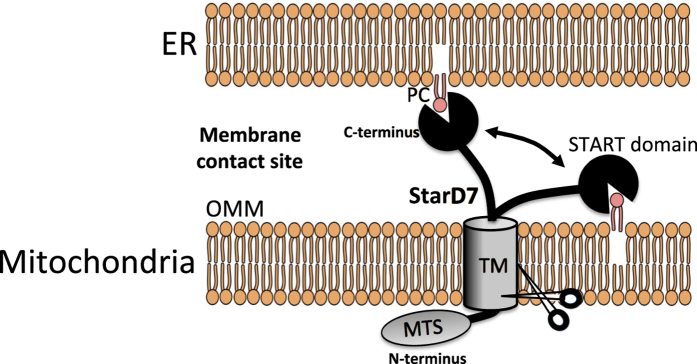
Scheme for the localization and function of StarD7 in the OMM. StarD7 is anchored in the OMM *via* its TM domain and extends its C-terminal START domain into the cytoplasm. At ER-mitochondria contact sites, the ER membrane comes close to the mitochondrial membrane through the action of tethering proteins. StarD7 may exchange/shuttle PC between the outer leaflet of the ER membrane and the outer leaflet of the OMM at the organelle contact sites. The scissors indicate a mitochondrial peptidase that cleaves the peptide bonds between Met^76^ and Ala^77^, and Ala^77^ and Ala^78^ within the TM domain to produce the mature form of the protein.

PC is the most abundant phospholipid in mitochondria. However, PC must be supplied to mitochondria from other PC-producing or PC-containing organelles because mitochondria lack the sequential enzymes necessary for PC synthesis. Our previous studies demonstrated that StarD7 specifically facilitates PC transfer to mitochondria, plays important roles in maintaining the PC content of mitochondrial membranes, and contributes to proper mitochondrial respiration and morphogenesis. However, from where StarD7 extracts PC and how StarD7 transfers PC to mitochondria is poorly understood. Here, we analyzed the intra-mitochondrial localization of StarD7, and demonstrated that StarD7 is inserted into the OMM through its N-terminal MTS and TM domains (Fig. 8). Thus, the START domain is exposed to the cytoplasmic face. Mitochondria are connected with the ER *via* tethering proteins such as MFN2, GRP75, Fis1-Bap31 and PTPIP51-VAPs, and therefore, the ER membrane may come sufficiently close to the mitochondrial membrane to initiate lipid transfer. StarD7 then extracts PC from the ER membrane *via* its cytoplasmic START domain and transfers it to the mitochondrial membrane. Conversely, StarD7 transports PC in the OMM to the ER membrane. This shuttling of PC by StarD7 would play an important role in maintaining the proper concentration of PC in the mitochondrial membrane. As the TM domain is conserved from nematodes to mammals, the molecular mechanism by which PC is transferred by StarD7 may be universal amongst animals. We also speculate that the release of StarD7 into cytosol means negative regulation of the activity because cytosolic StarD7 (StarD7-II) cannot rescue mitochondrial abnormalities in *StarD7*-KO cells^20^.

The mature form of StarD7 is integrated and/or associated onto the OMM, and also located in the cytosol in HEPA-1 and HepG2 cells. It is not fully understood how it is released to the cytosol. PTEN-induced putative kinase 1 (PINK1) is a well-studied protein that localizes in both the OMM and the cytosol. Similarly to StarD7, PINK1 has a MTS and a TM domain at the N-terminus. The TM domain is anchored in the OMM, and the C-terminal kinase domain faces the cytoplasm. When PINK1 precursor is imported into mitochondria, the peptide bond between Ala^103^ and Phe^104^ in the TM domain is cleaved to produce the mature form by presenilins-associated rhomboid-like protein (PARL), a rhomboid family protease which localizes in the inner mitochondrial membrane^22^. After PINK1 is cleaved by PARL, it is exported to the cytosol, driven by lateral diffusion^23^. Other mitochondrial proteases, such as m-AAA and ClpXP, are also involved in PINK1 cleavage^24^. In this study, we demonstrated that StarD7 is cleaved within the TM domain to produce the mature form (Fig. 7b). We found that ΔTM-V5 is mainly distributed in the mitochondria, and demonstrated that the TM domain is required for the cytosolic localization of StarD7 (Figs 2d,e and 5c,d). These results suggest that, similarly to PINK1, cleavage in the TM domain is necessary for the release of mature StarD7 from the OMM into the cytosol. However, MTS-TM is not sufficient for the release to cytosol, and a segment other than MTS-TM in StarD7 would be necessary because MTS-TM-GFP was not distributed in cytosol (Figs 4f and 5g). Interestingly, Saita *et al*. recently reported that StarD7 was identified as one of the protein substrates for PARL by proteomic approaches^25^. They also demonstrated that a peptide bond between Ala^77^ and Ala^78^ in the TM domain is cleaved by PARL. Here, in addition to the site described above, we found that a peptide bond between Met^76^ and Ala^77^ is also cleaved. It is possible that Ala^77^ is removed by aminopeptidase in cells or in the purification steps. It is also possible that StarD7 is cleaved by PARL at the both sites due to less recognition of cleavage specificity. Irrespective, the amino acid residues Met^76^, Ala^77^ and Ala^78^ have a very important role to be recognized as cleavage sites by PARL (Fig. 7d).

During the past several years, most LPTs that transport lipids between the ER and other organelles have been found to contain two directional domains; one for interaction with the ER^26^, and one for localizing in their specific organelles. For example, NIR2^27^, CERT^5, 28^, FAPP 2^4, 29^ and OSBP^30, 31^ transport PI, ceramide, glucosylceramide, and cholesterol/PI_4_P, respectively, between the ER and Golgi complex. All of these proteins have a FFAT (two phenylalanines in an acidic track) motif that can interact with the ER-localized VAP, and a PH domain that allows them to bind to PIPs on the Golgi. It is believed that these domains tether the ER and Golgi to form membrane contact sites, facilitating lipid transfer by these proteins between the organelles. In another example, long variant of ORP1 (ORP1L)^32, 33^ and StarD3^34, 35^ transport sterols between the ER and endosomes and both have a FFAT domain to interact with the ER and a domain for localization to the endosome. Unlike the LPTs described above, StarD7 lacks a motif such as FFAT for interacting with the ER-localized VAP. In this study, we demonstrated that part of StarD7 co-localized with MFN2, suggesting that StarD7 was distributed at the ER-mitochondria contact sites. We believe that a specific motif for interacting with the ER may not be necessary for StarD7 because the contact sites are formed by other proteins. It is important to identify which tethering proteins contribute to PC movement between ER and mitochondria. This will be further examined in a future study.

In summary, this study demonstrated that StarD7, a non-vesicular carrier of PC, is anchored onto the OMM *via* its N-terminal TM domain. The C-terminal START domain extends into the cytoplasm and is believed to capture and transfer PC between a mitochondrion and another organelle such as the ER at membrane contact sites.

## Methods

### Antibodies

The antibodies used in this study were: anti-V5 antibody purchased from Thermo Fisher Scientific (Waltham, MA), anti-TOM20 (612278) from BD Transduction Laboratories (San Jose, CA), anti-MFN2 (D2D10) and –SDHA (D6J9M) antibodies from Cell Signaling Technology (Danvers, MA), anti-GFP antibody (GF200) from Nacalai Tesque (Kyoto, Japan), Membrane Integrity WB Antibody Cocktail (anti-CVa, -Core1, -porin, -CypD, and -CytC antibodies) and anti-MTCO1 antibody (1D6E1A8) from Abcam (Cambridge, UK), anti-myc antibody (M192–3) from MBL (Nagoya, Japan), anti-actin from Sigma-Aldrich (St. Louis, MO), and anti-glyceraldehyde 3-phosphate dehydrogenase (GAPDH) antibody (5A12) from Wako Pure Chemicals (Osaka, Japan). Anti-StarD7 antibody was prepared as reported previously^14^.

### Construction of truncated StarD7 and chimeric GFP

Human StarD7 cloned in pcDNA3.1/V5-His-TOPO (WT-V5) was prepared as reported previously^14^. To construct ΔTM-V5, a truncated StarD7 lacking the TM domain between Arg^60^-Gln^96^, the DNA fragments encoding the amino acids (Met^1^-Gly^59^) and (Arg^97^-Ala^370^) were amplified by PCR using PrimeSTAR DNA polymerase (Takara Bio Inc., Shiga, Japan). For the (Met^1^-Gly^59^) and (Arg^97^-Ala^370^) fragments, two sets of primers, 5′-GGCATGCTCCCGCGGAGGCTGCT-3′(StarD7-Fwd) and 5′-GCTTCATCTCATTAATAGATCTGCCGAGGAGAACGCGGCGTG-3′ (StarD7-ΔTM-Rev) and 5′-CACGCCGCGTTCTCCTCGGCAGATCTATTAATGAGATGAAGC-3′ (StarD7-ΔTM-Fwd) and 5′-AGCATACTCAATCCGAGCCGG-3′(StarD7-Rev), respectively, were used. After gel purification, these DNA fragments were mixed and used as the PCR template to obtain full length ΔTM by PCR using one set of primers, hStarD7-Fwd and hStarD7-Rev. After adding a 3’A overhang using Taq polymerase, the amplified DNA fragment was cloned into pcDNA3.1/V5-His-TOPO vector according to manufacturer’s instructions.

To construct chimeric GFP fused with the MTS (Met^1^-Gly^59^) or MTS-TM domain (Met^1^-Gln^96^), the DNA fragments encoding the MTS or MTS-TM domain were amplified by PCR using two sets of primers, 5′-ATCTCGAGACATGCTCCCGCGGAGGCTG-3′ (StarD7-Fwd-XhoI) and 5′-ATGACTGCAGAGCGGCCGAGGAGAACGCGG-3′ (StarD7-MTS-Rev-PstI), and hStarD7-Fwd-XhoI and 5′-ATGACTGCAGACTGCAACTCCTCCTCCTGG -3′ (StarD7-MTS-TM-Rev-PstI), respectively. In each primer, the underlined sequence is the *Xho* I or *Pst* I recognition site. Amplified DNA fragments were digested with *Xho* I and *Pst* I, purified, then cloned into the *Xho* I/*Pst* I site of pEGFP-N3 (Clontech, Mountain View, CA).

For the construction of StarD7 fused with myc-tag at the C-terminus, the DNA fragment encoding the protein was amplified using one set of primers, 5′-ATAAGCTTCCATGCTCCCGCGGAGGCTGCT-3′ (StarD7-Fwd-HindIII) and 5′-ATCTCGAGAGCATACTCAATCCGAGCAGG-3′ (StarD7-Rev-XhoI), and cloned into the *Hind* III/*Xho* I site of pcDNA3-C-Myc. The underlined sequence is the *Hind* III or *Xho* I recognition site.

The sequences of the cloned DNA fragments were confirmed by sequencing using an ABI PRISM 310 Genetic Analyzer (Applied Biosystems, Foster City, CA) and a BigDye Terminator v1.1 Cycle Sequencing Kit (Applied Biosystems).

### Cell culture, expression, and immunocytochemistry

Mouse HEPA-1 and human HepG2 cells, derived from hepatocellular carcinoma, were cultured in DMEM (high glucose) with 10% FBS at 37 °C in a humidified incubator containing 5% CO_2_. Cells were transfected with the expression vectors using Lipofectamine 2000 (Thermo Fisher) according to the manufacturer’s instructions. Twenty-four hours after transfection, the cells were treated with 250 nM MitoTracker Red CMXRos (Thermo Fisher) and incubated at 37 °C for 30 min. Then, cells were fixed with 4% paraformaldehyde in PBS for 15 min, washed with PBS, permeabilized with 0.005% digitonin (w/v) (Sigma-Aldrich) or 0.1% Triton X-100 (w/v) for 10 min, then blocked with 5% skim milk for 30 min. Cells were then incubated with primary antibodies overnight at 4 °C, followed by washing and immunostaining with fluorescently labeled secondary antibodies conjugated with Alexa 488 or 594 (Thermo Fisher) for 1 h at room temperature. Nuclei were stained with DAPI. Samples were observed with a confocal microscope (FV10i; Olympus, Tokyo, Japan or LSM780; Zeiss, Oberkochen, Germany).

### Isolation of mitochondria, proteinase K treatment, and alkaline carbonate extraction

Mitochondria and cytosolic fractions were freshly prepared from cells plated at 60–70% confluent using a Mitochondria Isolation Kit for Cultured Cells (Thermo Fisher) according to the manufacturer’s instructions. For proteinase K digestion, mitochondria were resuspended in isotonic mitochondrial buffer (10 mM Hepes buffer, pH 8.0, 250 mM sucrose, 0.5 mM EGTA) and incubated with different concentrations of proteinase K (Thermo Fisher) for 30 min on ice. Digestion was terminated with 10% trichloroacetic acid (w/v). After centrifugation, the protein pellet was washed with acetone, dissolved in sample buffer, then the proteins were separated by SDS-PAGE and analyzed by Western blotting. Alkaline carbonate extraction was performed by suspending the mitochondria in 0.1 M sodium carbonate buffer, pH 11.5, and incubating on ice for 30 min. Membrane pellets and supernatants were separated by centrifugation at 100,000 × g for 30 min at 4 °C. Components in the supernatant were precipitated by adding 10% trichloroacetic acid. The membrane and soluble fractions were dissolved in the same volume of sample buffer (65 μl), then the same volume of protein solutions (15 μl) were separated by SDS-PAGE and analyzed by Western blotting.

### Western blotting analysis

Western blotting was performed by separating the proteins with SDS-PAGE, transferring the proteins to nitrocellulose membranes (Amersham Protran, GE Healthcare, Chicago, IL) using a Trans-Blot SD Semi-Dry Transfer blotter (Bio-Rad Laboratories, Hercules, CA), then incubating the membranes with 5% (w/v) skim milk in TBS for 1 h and washing three times with T-TBS (TBS containing 0.02% Tween 20). Membranes were then incubated with primary antibodies overnight at 4 °C, washed three times with T-TBS, then incubated with horseradish peroxidase–conjugated IgGs for 1 h at room temperature. Membranes were washed three times with T-TBS and stained with Clarity Western ECL Substrate (Bio-Rad) according to the manufacturer’s instructions and visualized using a ChemiDoc MP (Bio-Rad).

### Rescue experiment

*StarD7*-knockout (KO) HEPA-1 cells were prepared as described previously^20^. *StarD7*-KO cells (designated KO2 in the previous study) were transfected with a plasmid encoding WT-V5 or ΔTM-V5 using Lipofectamine 2000 according to the manufacturer’s instructions, then cultured in high-glucose DMEM for 3 days. Cells were lysed in 20 mM Tris-HCl buffer (pH 8.0) containing 0.5% Triton X-100, 1 mM EDTA, 0.5 mM PMSF, and 5 µg/ml leupeptin, antipain, and chymostatin. Proteins (18 μg) were separated by SDS-PAGE, and the protein levels of MTCO1, V5-tagged StarD7, and actin were analyzed by Western blotting. Protein band intensity was measured using Quantity One software (Bio-Rad). Data were analyzed in the linear range of the signal intensity, and the band intensity was normalized against that of actin.

To establish *StarD7*-KO cells stably expressing WT or ΔTM, cells were transfected with WT or ΔTM cloned into a pCAG vector^36^ using Lipofectamine 2000, and cultured for 2 weeks with 3 mg/ml G418. Resistant clones showing protein expression were used for the quantification of mitochondrial PC.

### Isolation of mitochondria and quantification of PC by LC/MS/MS

Mitochondria were isolated from cells using a hybrid Percoll-metrizamide gradient method described by Storrie *et al*.^37^. We substituted Nycodenz for metrizamide because the latter was difficult to obtain as described in ref. 20. Phospholipids were extracted from purified mitochondria (50 µg of protein) according to the Bligh and Dyer method^38^ in the presence of 2 µg of internal standards (1,2-dipentadecanoyl PC; Avanti Polar Lipids, Alabaster, AL). Lipids were analyzed by reverse-phase ultra-high-pressure liquid chromatography using an Acquity UPLC BEH C18 column (1.7 μm, 2.1 × 50 mm) (Waters, Milford, MA) coupled to a 5500 QTRAP mass spectrometer (Sciex Inc., Framingham, MA) as described in ref. 20. PC was quantified using MultiQuant, version 2.0 (Sciex), and normalized against the internal standards.

### Identification of the cleavage site of StarD7 by LC/MS/MS

HEPA-1 cells were transfected with a plasmid encoding human StarD7 fused with a 3 × Flag tag at the C-terminus. After lysing the cells, StarD7 was affinity purified using anti-Flag antibody conjugated to beads (Sigma-Aldrich). Proteins were eluted from the beads with 3 × Flag peptide (Sigma-Aldrich) and separated by SDS-PAGE. Protein bands corresponding to the mature form of StarD7 (37 kDa) were excised, reduced with 10 mM dithiothreitol, alkylated with 50 mM iodoacetamide, and digested with 10 ng/μl trypsin overnight at 37 °C. Peptides were extracted from the gel by incubation with 50% acetonitrile (v/v) containing 1% formic acid, then the extracts were dried in a SpeedVac. For desalting, samples were dissolved in 0.1% formic acid, applied to a GL-Tip SDB (GL Sciences, Tokyo, Japan), and eluted with 80% acetonitrile (v/v) containing 0.1% formic acid. For LC/MS/MS experiments, samples were analyzed using a TripleTOF 6600 mass spectrometer (SCIEX, Framingham, MA) coupled to a nanoLC Eksigent 400 system comprising a reverse-phase LC with a nano column (75 μm × 15 cm ChromXP C18-CL, 3 μm, 120 Å). The MS and MS/MS spectral data were collected using Analyst software (SCIEX), and peptides were identified using protein plot software (SCIEX).

### Site-directed mutagenesis

Mutants of StarD7 were generated using an inverse PCR-based site-directed mutagenesis method and PrimeSTAR DNA polymerase with StarD7 plasmid as the template. Amino acids around the putative cleavage site (Arg^68^, Ala^72^, Ser^73^, Ala^74^, Leu^75^, Met^76^, Ala^77^, Ala^78^, Leu^79^, or Ala^80^) were individually changed to aspartic acid. The rationale for the substitution to aspartic acid is, (1) it is not a helix breaker, (2) the Asp substitution is expected to significantly influence on the protein proccessing because there are no acidic amino acids between Agr^68^-Ala^80^, and (3) Ala-scanning is not proper because there are five alanine residues there. After digestion with *Dpn*I, PCR products were ligated with ligase mix (Ligation High; TOYOBO, Osaka, Japan) in the presence of T4 polynucleotide kinase (Takara Bio Inc.).

## Electronic supplementary material

Supplementary information
