# Supplementary material for: Identification of the N-terminal transmembrane domain of StarD7 and its importance for mitochondrial outer membrane localization and phosphatidylcholine transfer
Source: Sci Rep. 2017 Aug 18;7:8793. doi: 10.1038/s41598-017-09205-1 (PMC5562819; doi:10.1038/s41598-017-09205-1)
Supplement: Supplementary file 1 — Supplementary information [file 41598_2017_9205_MOESM1_ESM.pdf]

Supplemental Information

Identification of the N-terminal transmembrane domain of StarD7 and its importance for mitochondrial outer membrane localization and phosphatidylcholine transfer

Yasuhiro Horibata, Hiromi Ando, Motoyasu Satou, Hiroaki Shimizu, Satomi Mitsuhashi, Yasuo Shimizu, Masahiko Itoh and Hiroyuki Sugimoto

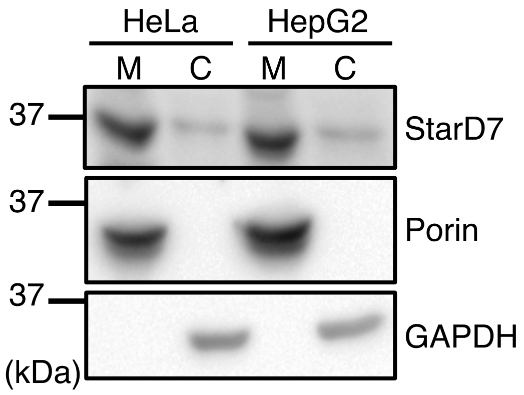

FIGURE. S1. Distribution of endogenous StarD7 in the mitochondria and cytosol of HeLa and HepG2 cells.

Mitochondria and cytosol were separated from HeLa or HepG2 cells by subcellular fractionation and analyzed by western blotting using anti-StarD7, -porin, and -GAPDH antibodies. M and C indicate mitochondria and cytosol, respectively.

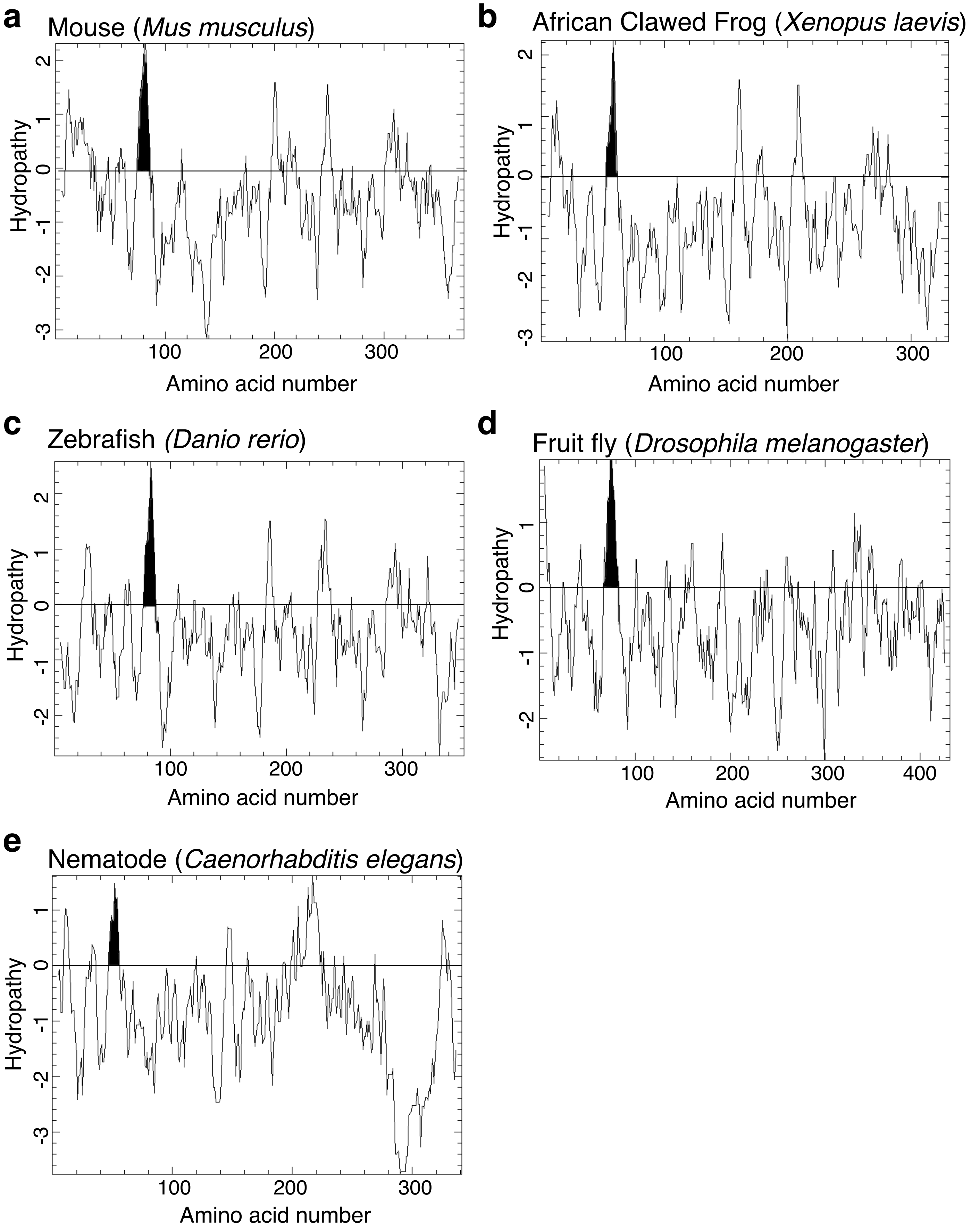

FIGURE. S2. Hydrophobicity plots of StarD7 isolated from various animals.
Amino acid sequences of mouse (a), frog (b), zebrafish (c), fruit fly (d), and nematode (e) were analyzed by Kyte-Doolittle hydrophobicity plots. Each putative TM domain is indicated by *black shading under the peak*.

a

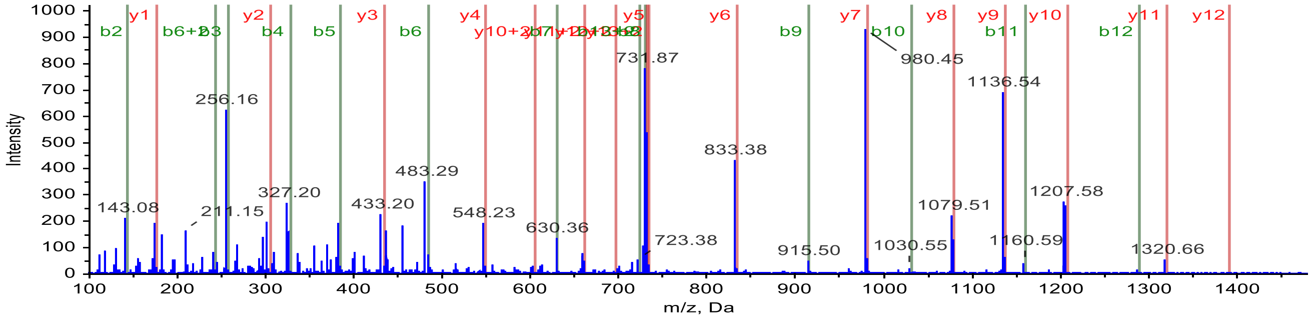

b

Residue	b	b+2	y	y+2		
A	72.0444	36.5258	1462.7325	731.8699	b1	y13
A	143.0815	72.0444	1391.6954	696.3513	b2	y12
L	256.1656	128.5864	1320.6583	660.8328	b3	y11
A	327.2027	164.105	1207.5742	604.2907	b4	y10
G	384.2241	192.6157	1136.5371	568.7722	b5	y9
V	483.2926	242.1499	1079.5156	540.2615	b6	y8
F	630.361	315.6841	980.4472	490.7272	b7	y7
V	729.4294	365.2183	833.3788	417.193	b8	y6
W	915.5087	458.258	734.3104	367.6588	b9	y5
D	1030.5356	515.7715	548.2311	274.6192	b10	y4
E	1159.5782	580.2928	433.2041	217.1057	b11	y3
E	1288.6208	644.8141	304.1615	152.5844	b12	y2
R	1444.7219	722.8646	175.119	88.0631	b13	y1

c

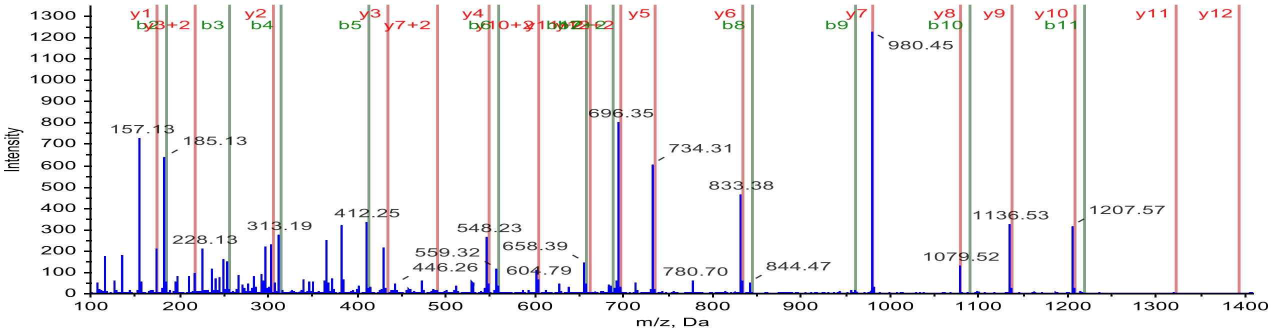

d

Residue	b	b+2	y	y+2		
A	72.0444	36.5258	1391.6954	696.3513	b1	y12
L	185.1285	93.0679	1320.6583	660.8328	b2	y11
A	256.1656	128.5864	1207.5742	604.2907	b3	y10
G	313.187	157.0972	1136.5371	568.7722	b4	y9
V	412.2554	206.6314	1079.5156	540.2615	b5	y8
F	559.3239	280.1656	980.4472	490.7272	b6	y7
V	658.3923	329.6998	833.3788	417.193	b7	y6
W	844.4716	422.7394	734.3104	367.6588	b8	y5
D	959.4985	480.2529	548.2311	274.6192	b9	y4
E	1088.5411	544.7742	433.2041	217.1057	b10	y3
E	1217.5837	609.2955	304.1615	152.5844	b11	y2
R	1373.6848	687.3461	175.119	88.0631	b12	y1

FIGURE. S3. Determination of the cleavage site in the TM domain of StarD7 using LC/MS/MS.

MS/MS spectra and masses of the amino acid residues of Ala^{77}-Arg^{89} (a and b) and Ala^{78}-Arg^{89} (c and d) peptides, respectively.

Fig. 1c

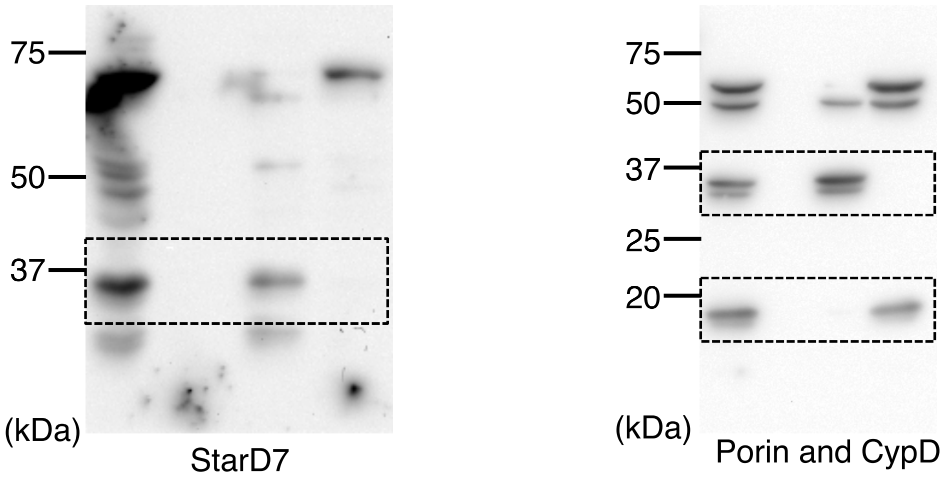

Fig. 1d

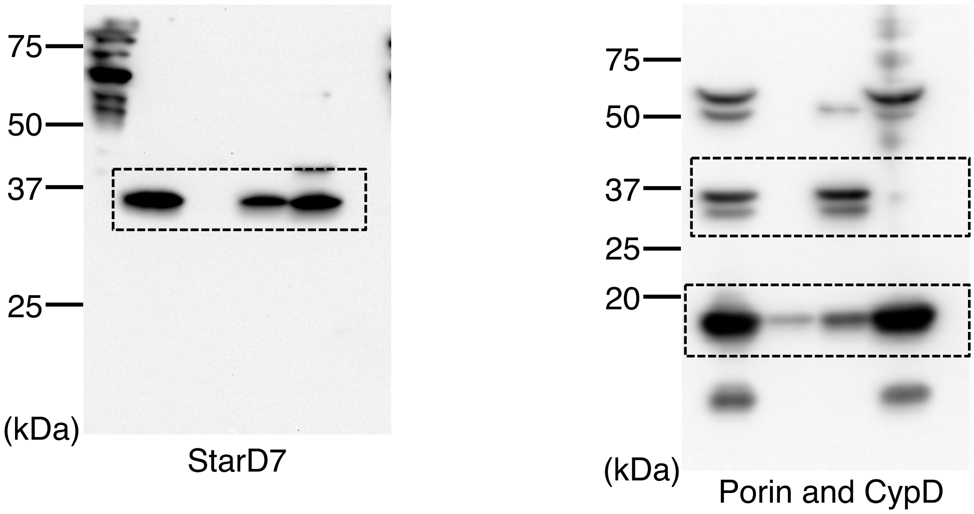

Fig. 2b and 2c

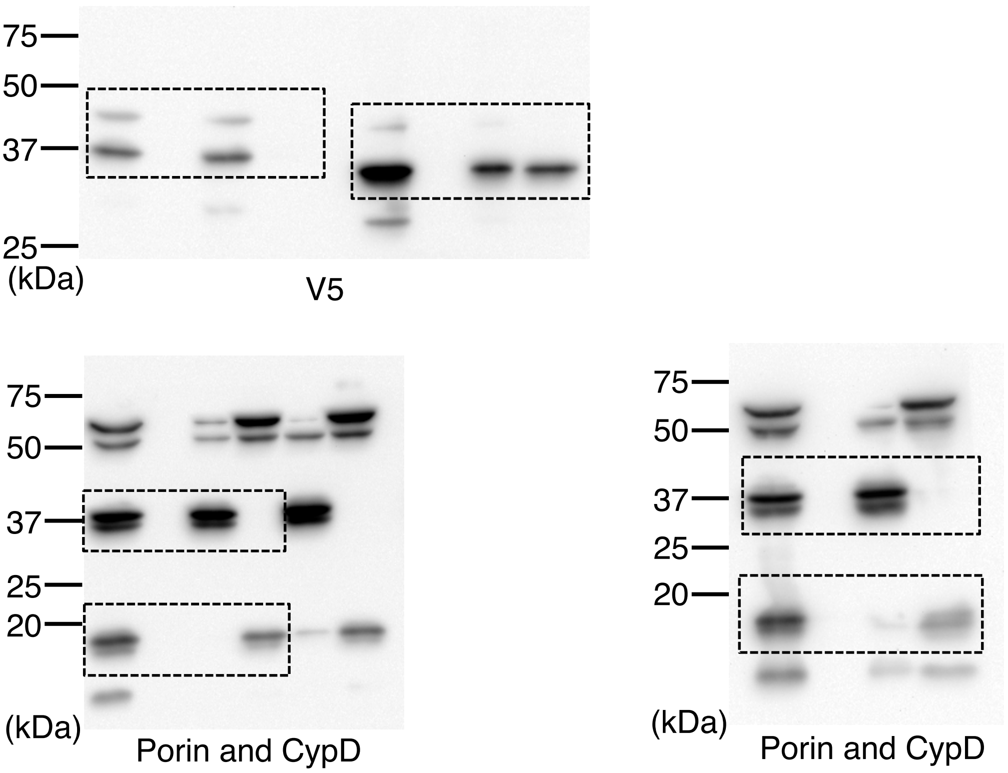

FIGURE. S4. Unprocessed Western blots in figures and supplemental figures

Fig. 2e

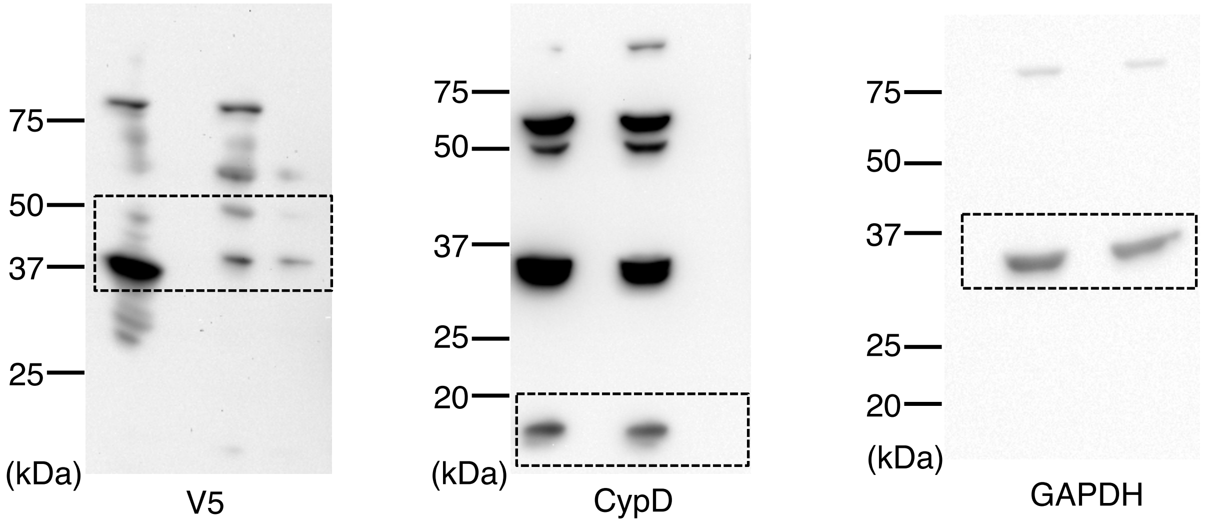

Fig. 3a

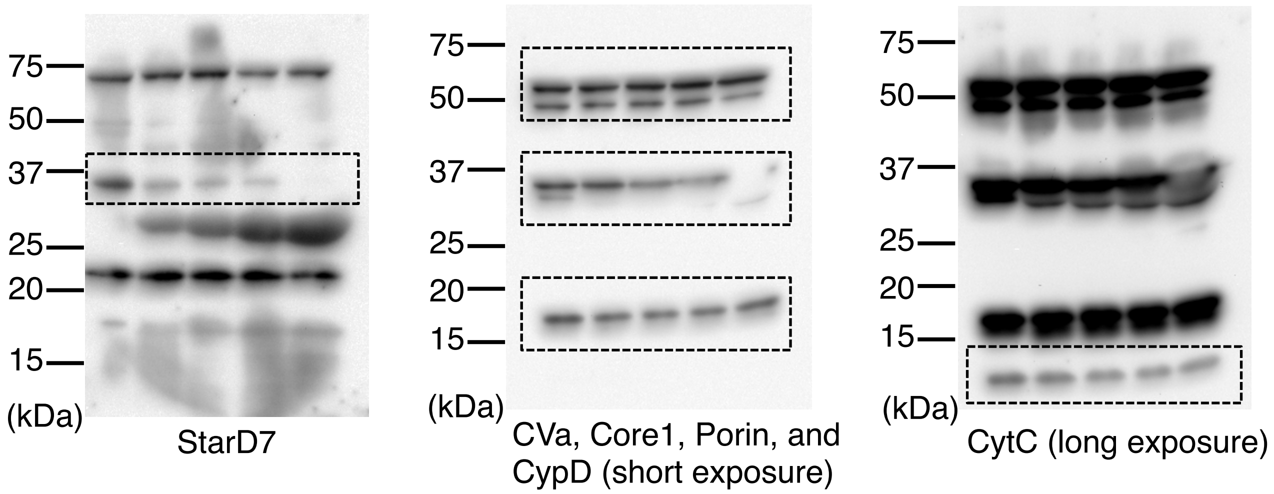

Fig. 3b

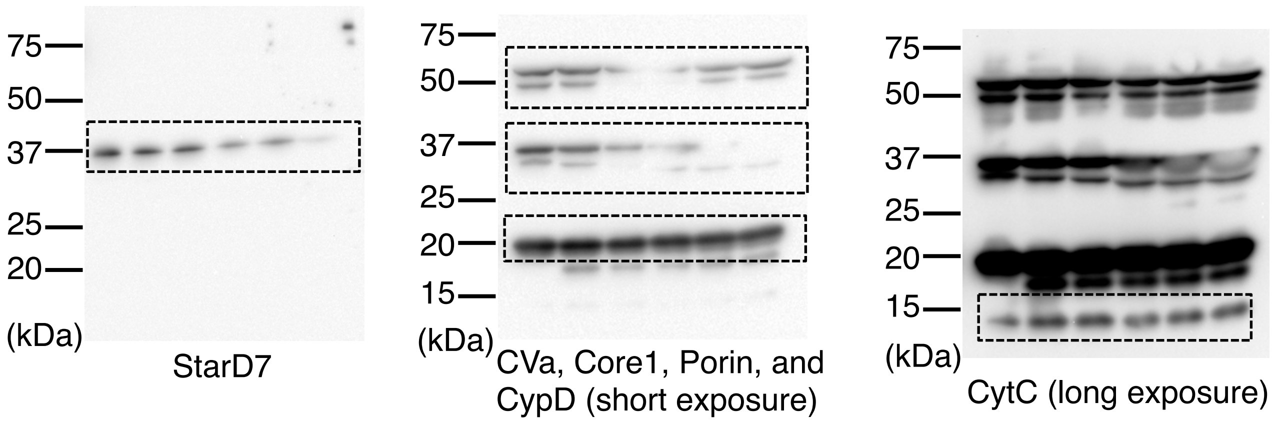

FIGURE. S4. Continued

Fig. 3c and 3d

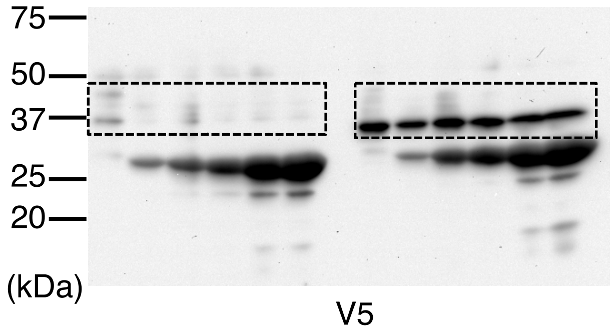

V5

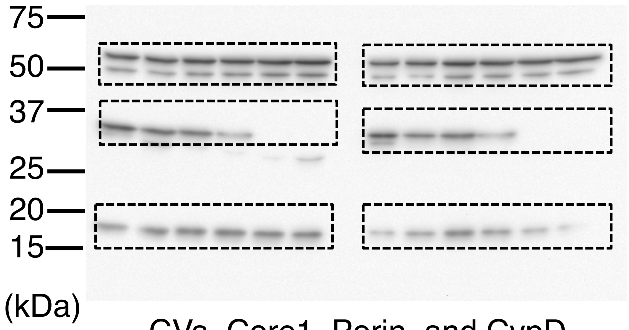

CVa, Core1, Porin, and CypD
(short exposure)

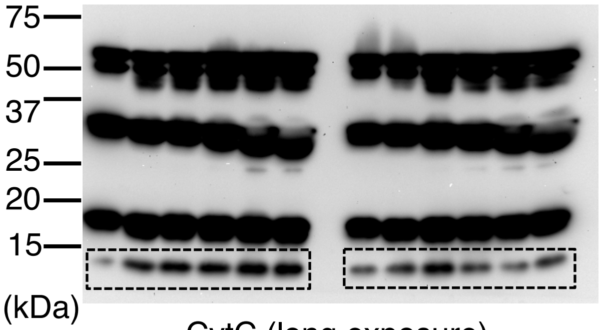

CytC (long exposure)

Fig. 4b and 4c

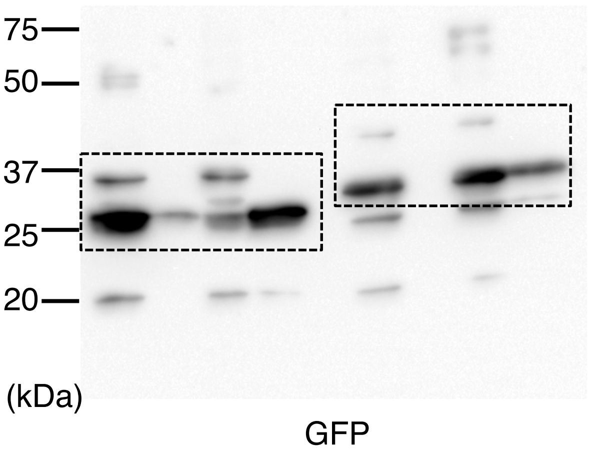

GFP

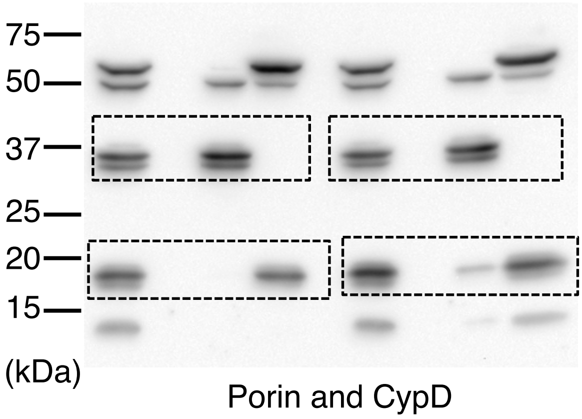

Porin and CypD

FIGURE. S4. Continued

Fig. 4d and 4e

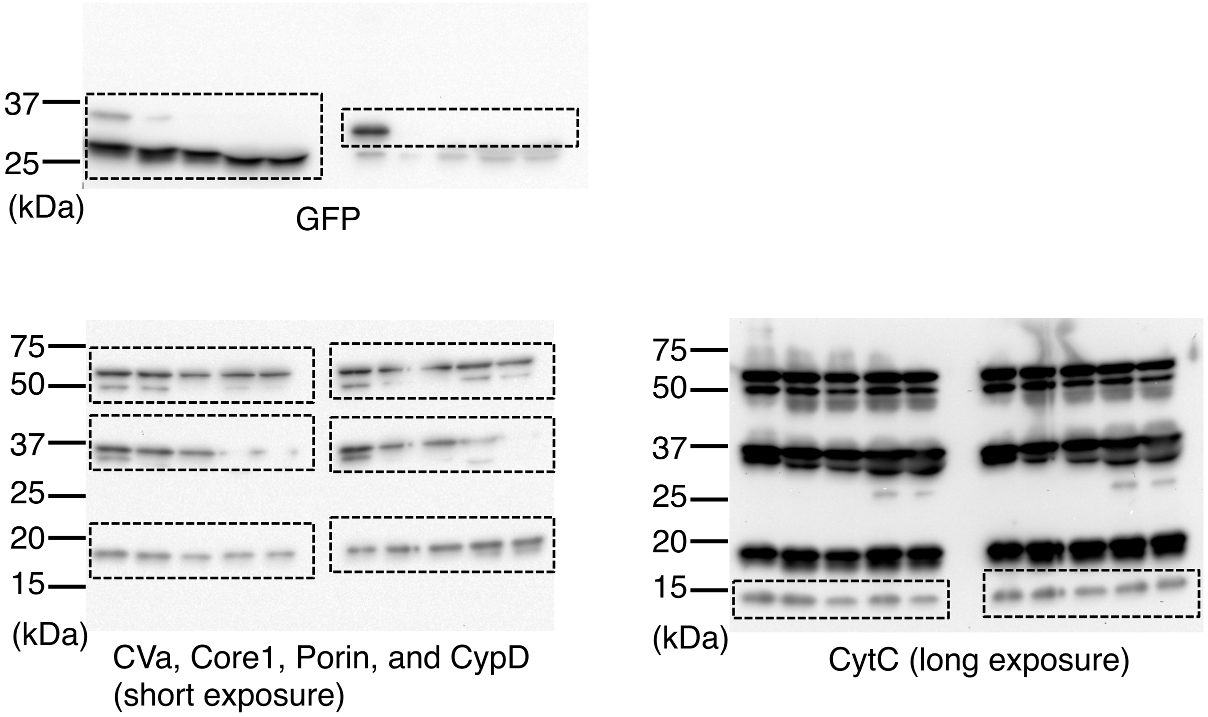

Fig. 4f

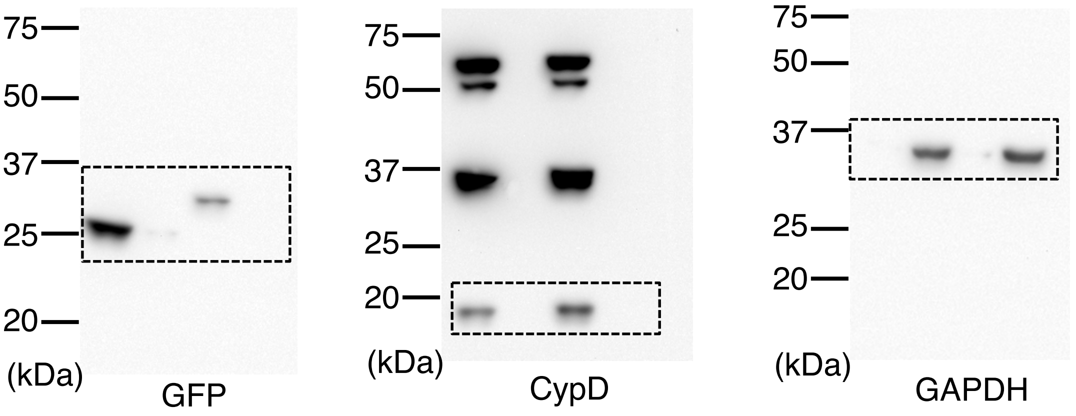

Fig. 6a

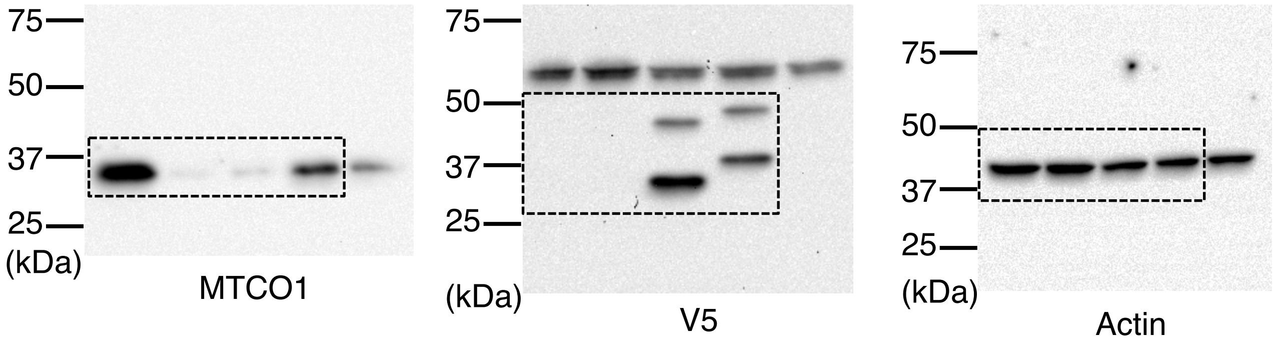

FIGURE. S4. Continued

Fig. 6c

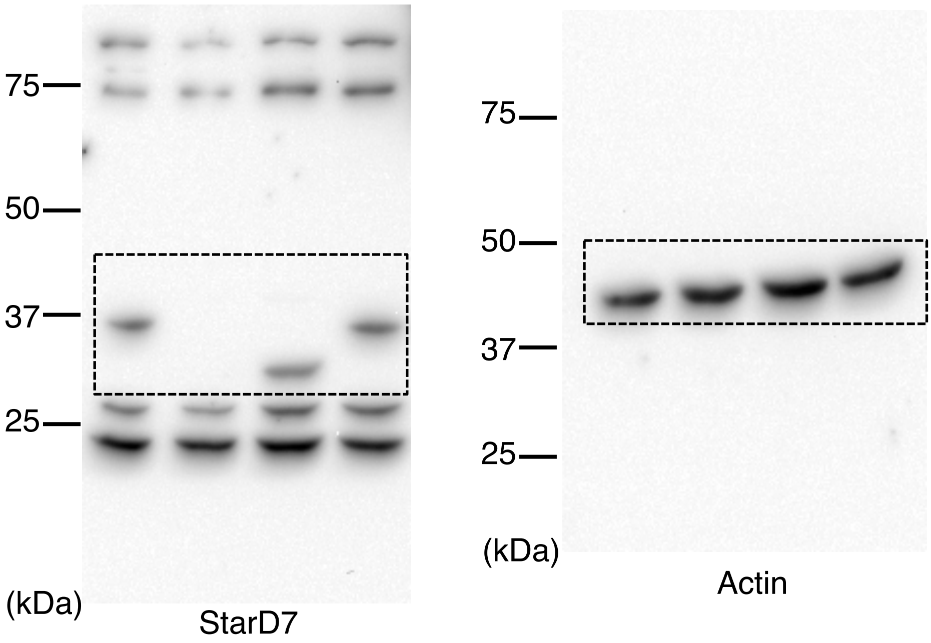

Fig. 7d

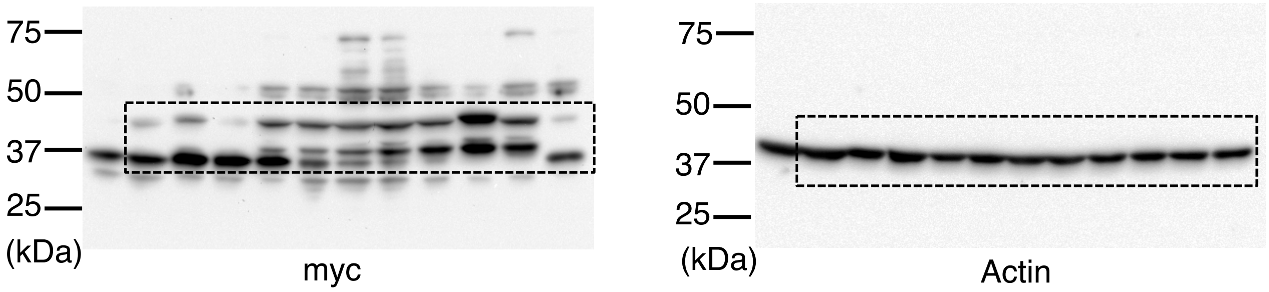

Fig. S1

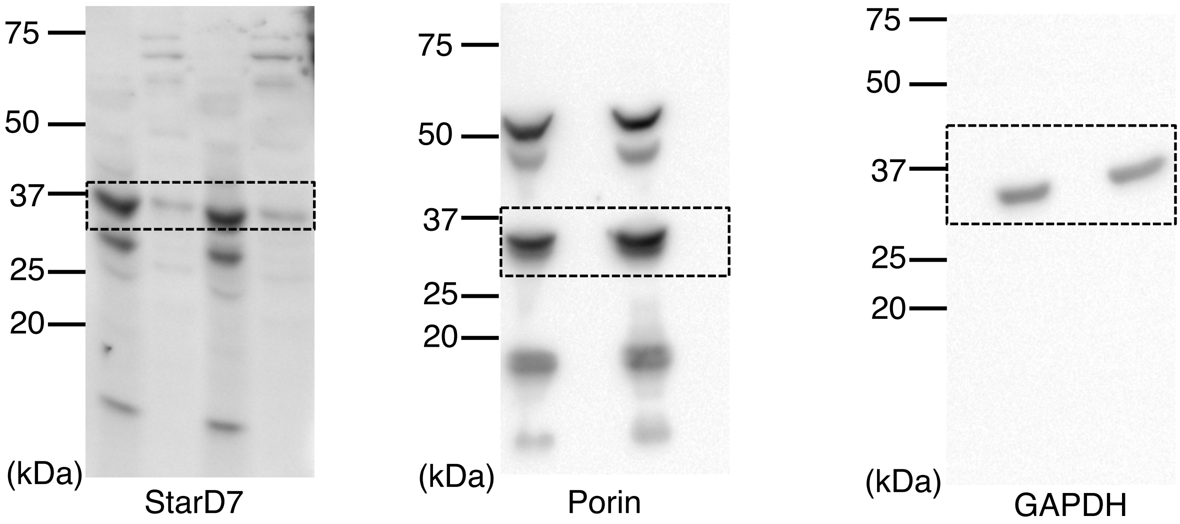

FIGURE. S4. Continued
